# The importance of a common global health definition: How Canada’s definition influences its strategic direction in global health

**DOI:** 10.7189/jogh.02.010301

**Published:** 2012-06

**Authors:** Ruth M. Campbell, Maja Pleic, Hillary Connolly

**Affiliations:** 1 Wilson Centre, Toronto General Hospital, Toronto, Canada; 2 Harvard Global Equity Initiative, Cambridge, MA, USA; 3 Centre for Addiction and Mental Health, Toronto, Canada

In late 2011, the Expert Panel on Canada’s Strategic Role in Global Health (herein: the panel) selected a global health definition for Canada. This decision is significant as the chosen definition forms the foundation of the panel’s forthcoming recommended strategic role for Canada in global health. In turn, the global health community can draw lessons from Canada’s decision to inform their own understanding of the term and demystify priority setting. In this paper we examine the five definitions considered by the panel and analyze the core characteristics of each in order to understand the rationale for the final choice as well as the implications of the chosen definition. To understand the basis upon which Canada will build its strategic direction for global health it is useful to frame this analysis in light of the other short-listed definitions.

## WHY IS THE DEFINITION OF GLOBAL HEALTH IMPORTANT?

There has been a tremendous amount of discussion about global health without rooting the term itself to a common definition. Countless books and journal articles have been written and university programs have been designed around global health without a definition of the term. There are numerous examples of work being done in this field without a clear definition in place [1,2]. Indeed, it is often not clear how people and organizations engaged in global health are using the term. An analogy would be for a medical team to discuss an intervention for a patient with condition ‘x’, without an agreed-upon definition of the condition itself. Because global health is composed of, and relies on, multiple disciplines and sectors of society – which work from different languages, values, motivations and perspectives – it is important that at the very least there be a clear communication of what each actor is referring to when they use the term global health. For actors to write, instruct or develop meaningful strategies for global health, they require a definition of global health. This definition can be used as a frame from which to work and can be communicated to others.

## BACKGROUND

Current global health trends, including epidemiological and demographic transitions, the rising burden of disease, climate change and the increasing awareness of global disparities in health, have heightened interest in the field of global health among the international medical and public health communities [3-6]. Yet, there is a considerable amount of ambiguity and controversy about what ‘global health’ means [7,8].

In September 2010, the Canadian Academy of Health Sciences, with the assistance of the Council of Canadian Academies, brought together 15 Canadian global health experts to form the Expert Panel on Canada’s Strategic Role in Global Health [9,10]. The panel was tasked with assessing whether Canada ought to play a more strategic role in global health and, if so, to identify potential roles [9]. According to the Canadian Academy of Health Sciences, “Canada does not have a national multi-sectoral strategy to address the increasingly complex issue of global health” [9]. In order to frame deliberations about potential strategic roles for Canada in global health, the expert panel felt a common definition of global health was necessary [9,11]. Each of the five definitions had been widely disseminated in leading peer-reviewed health journals or had been developed by key actors in the research and practice of global health [11].

In the November 2011 report, *Canadians Making a Difference*, the panel indicated the Koplan et al. (2009) definition was agreed upon as the common definition for global health (**Table 1**) [9,14]. The purpose of this essay is to outline an approach for evaluating global health definitions in order to ultimately select the most appropriate definition. In this paper we analyze the five definitions short-listed by the panel of Canadian global health experts [10], we deconstruct the characteristics of each and consider the implications on strategic priorities and initiatives of including or excluding these characteristics in a definition of global health.

**Table 1:** Inductive analysis of global health definitions

Brown: “Global health” in general, implies consideration of the health needs of the people of the whole planet above the concerns of particular nations. The term “global” is also associated with the growing importance of actors beyond governmental or intergovernmental organizations and agencies.[12]								
Primary characteristics					Secondary characteristics			
Equity	Global conceptualization	Causes	Means	Solutions	Source of obligation	Multi-disciplinary	Actors	Reactive (R) Proactive (P)
No	Yes	No	No	No	No	No	Yes	No

European Commission: Global health… is about worldwide improvement of health, reduction of disparities, and protection against health threats. [13]								
Primary characteristics					Secondary characteristics			
Equity	Global conceptualization	Causes	Means	Solutions	Source of obligation	Multi-disciplinary	Actors	Reactive (R) Proactive (P)
Yes	Yes	No	No	No	No	No	No	P

Koplan: Global health is an area of study, research, and practice that places a priority on improving health and achieving equity in health for all people worldwide. Global health emphasizes transnational health issues, determinants, and solutions; involves many disciplines within and beyond the health sciences and promotes interdisciplinary collaboration; and is a synthesis of population-based prevention with individual-level clinical care. [14]								
Primary characteristics					Secondary characteristics			
Equity	Global conceptualization	Causes	Means	Solutions	Source of obligation	Multi-disciplinary	Actors	Reactive (R) Proactive (P)
Yes	Yes	Yes	Yes	Yes	No	Yes	No	R+P

United Kingdom: Global health refers to health issues where the determinants circumvent, undermine or are oblivious to the territorial boundaries of states, and are thus beyond the capacity of individual countries to address through domestic institutions. Global health is focused on people across the whole planet rather than the concerns of particular nations. Global health recognizes that health is determined by problems, issues and concerns that transcend national boundaries.[15]								
Primary characteristics					Secondary characteristics			
Equity	Global conceptualization	Causes	Means	Solutions	Source of obligation	Multi-disciplinary	Actors	Reactive (R) Proactive (P)
No	Yes	Yes	No	No	Yes	No	No	No

U. S. Institute of Medicine: Global health is the goal of improving health for all people in all nations by promoting wellness and eliminating avoidable disease, disability, and death. It can be attained by combining population-based health promotion and disease prevention measures with individual-level clinical care.[16]								
Primary characteristics					Secondary characteristics			
Equity	Global conceptualization	Causes	Means	Solutions	Source of obligation	Multi-disciplinary	Actors	Reactive (R) Proactive (P)
Yes	Yes	No	Yes	Yes	No	No	No	R + P

The characteristics of each definition were identified by inductive analysis, which allows characteristics to emerge from patterns found in the definitions being examined without presupposing what these characteristics will be [17]. We read and analyzed the definitions independently in order to identify distinct characteristics and to consider their role in a definition of global health. Consensus on the characteristics was reached through group discussion of both the definitions and potential examples of the different characteristics. The definitions were then coded for the occurrence or non-occurrence of each characteristic [11].

We determined whether a characteristic is primary or secondary by examining how it is portrayed in the literature. Primary characteristics are those that the global health grey and peer-reviewed literature portray as essential to a concept of health that is differentiated as global. Without these characteristics, the term global health would cease to be distinct from other areas of health or it would be too vague to be actionable. Secondary characteristics are those mentioned in the literature that add detail or fine-tune the concept but are not regarded as crucial to the distinctiveness of global health or necessary for clarity.

## PRIMARY AND SECONDARY CHARACTERISTICS OF GLOBAL HEALTH DEFINITIONS

Through the initial inductive analysis of the definitions, we identified five primary characteristics and four secondary characteristics overall (**Table 1**).

### Primary characteristics

The five characteristics considered *primary* are noted in the left hand side of **Table 1**.

#### 1. Equity

The book, *Global Health and Global Health Ethics,* states that “the most striking feature about the state of global health is that it is characterized by such radical inequities” [7]. Indeed, basic statistics on inequities in health status and access provide the background of global health work. The lifetime risk for a Canadian woman dying from pregnancy complications or childbirth is 1 in 11 000 [7]; the lifetime risk for a woman in Niger is 1 in 7 [7]. Similarly, life expectancy at birth varies by over 50% depending on the country of birth. For those born in Canada or Japan, the average life expectancy is 80 years or more, whereas in Afghanistan and Sierra Leone life expectancy is approximately 40 years [7]. While a Canadian child diagnosed with acute lymphoblastic leukemia has a 90% chance of being cured; in the poorest countries of the world more than 90% of children diagnosed with this disease will die [18]. This is the context in which global health is practiced today.

Faced with such appalling disparities, much of global health research and practice is based on the underlying notion of equity [19,20]. In the past, international health focused on understanding “the other” or “the tropical” and was largely shaped in the context of colonialism. Today, the forces of globalization and the information and communication revolution have brought glaring global health disparities into full view and are the lens through which much of global health work is done. As a result, ‘equity’ was listed by the expert panel as the first of three core principles that will guide the global health strategic vision for Canada, along with effectiveness and engagement [9]. Thus, it is telling that the expert panel chose a definition that not only includes the principle of ‘equity’, but one that emphasizes it in the very first sentence.

#### 2. Global conceptualization

A global conceptualization, differentiable from an international or supra-national perspective, is an integral component of a global health definition [21]. ‘Global’ health goes beyond the nation and focuses instead on vulnerable populations worldwide [8]. The difference between the terms international and global may appear small at first, however, the implications are profound and at the very heart of the practice of global health and therefore also its definition. While international refers to nations interacting with nations, and supranational suggests bodies above the national level, global implies ignoring borders altogether and bridging gaps between need and care wherever they may exist. This is not to say that borders are porous or nations unimportant. National governments continue to provide the bulk of funding for development assistance in health, although the channels through which they are funneled are increasingly becoming global actors – such as the Global Fund and the Bill and Melinda Gates Foundation [22]. What is truly ‘global’ is the conceptualization of health itself, represented by the goal of health for all people, irrespective of location or nationality. Not surprisingly, all five of the definitions considered by the expert panel embrace a global conceptualization and refer to the goal of “health of all people” or health for “people worldwide”.

#### 3. Causes

Causes are the contextual factors that determine a health issue. Causes can include the social, economic and physical environment, as well as individual characteristics and behaviors [23]. This characteristic highlights the fact that global health is not only a field of study or practice but also a response to a burgeoning set of upstream challenges. Understanding the causes of these challenges is crucial for addressing global health status, and also for distinguishing its practice from humanitarian aid. Many of the biggest global health challenges are intimately tied to socio-political and economic forces related to resources, i.e. famines; health infrastructure and skilled worker shortages; and access barriers to essential medicines, vaccines and health services. Thus to ignore the causes of global health challenges – in the field and in the definition – is to miss the very reason for the existence of the field. The challenges and disparities in global health are the *raison d’ętre* for the research, study and practice of global health. Unlike a science or an art, the field of global health is very much about providing solutions to current problems. As such, it would be short-sighted not to consider the causes of global health problems in order to better formulate the solutions. The causes ought to be included in a comprehensive and complete definition of the field. Specifying causes narrowly or broadly will either focus or widen the scope of factors to be addressed in global health activities.

**Figure:**
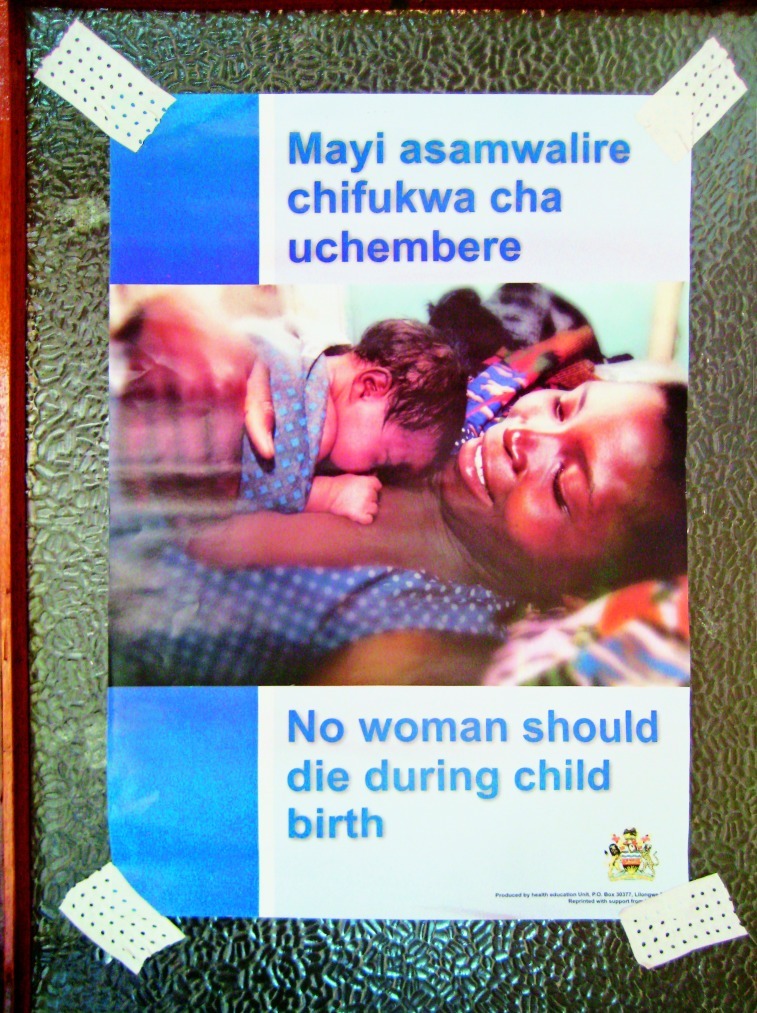
Photo: Courtesy of Alasdair Campbell, personal collection

#### 4. Means

Means are the methods and paths through which health activities take place. Depending on the conceptualization of global and the scope of causes, the means may range from providing individual-level clinical care or community education, through population-level disease prevention, to large scale national or international interventions [8]. Specifying the means pushes the concept from descriptive to prescriptive, or from ‘what’ to ‘how’ global health ‘should’ be carried out. Global health is not only a field of study but also a field of practice. Excluding *means* from the definition of global health would render it incomplete.

#### 5. Solutions

Solutions are the activities undertaken to address health issues. The range of potential solutions varies with the extent of available resources, political will, time frame and scope of goals [24,25]. Solutions to global health challenges can, and often do, come from multiple sectors of society including the public system, academia, civil society, and the private sector. Specifying global health solutions will guide priority setting for resource allocation under global health initiatives [26]. Decisions about solutions addressing imminent health problems will take precedence over investing in future health system capacity or tackling determinants of health [27,28]. Global health is about understanding the *causes* and finding the *means* to provide *solutions* to the challenges and disparities in health status of people worldwide. Thus solutions are a crucial component of a global health definition because they signal the fact that global health is not just a study or a practice, but a means to an end goal: the end of unnecessary, preventable and treatable inequities in global health status. Without including the solutions in the definition, the field and the definition lose the glue that holds it all together since there would be little use of studying or practicing global health, with its accompanying disparities and challenges, if global health practitioners were not interested in providing the solutions to said challenges. Thus we consider *solutions* an essential component of a global health definition.

### Secondary characteristics

The four characteristics considered *secondary* are noted in the right hand side of **Table 1**.

#### 1. Source of obligation

One of the examined definitions [15] refers to the source of obligation for global health activity. Specifically, resource-rich entities are obligated to help those with fewer resources tackle their health problems. This characteristic is part of the extensive ethical discussion on obligations [7], as the source and nature of obligations have implications for conceptualizations, means and solutions in global health. Nevertheless, for a working definition, obligation is adequately reflected in the primary characteristics. Specifically, the motive of global health is reflected in the shared desire to find solutions to challenges; while the feelings of or sources of obligation will undoubtedly vary across individuals and actors and cannot be summed up for the entire field and all those who practice or study it.

#### 2. Multidisciplinary approach

The primary characteristics of a global health definition - that it crosses borders, has a multitude of causes and involves a range of means and solutions – implies the need for multiple professionals and disciplines in addition to medical professionals [29]. Although many global health issues require a multidisciplinary approach, (for example, access to affordable antiretroviral treatments or implementing tobacco control strategies) it need not necessarily be so. Further, involving multiple disciplines all the time may not be necessary or efficient. A multidisciplinary approach is often, but not always, needed and beneficial and is therefore not an essential component of the field of the definition.

#### 3. Actors

Typically, global health issues are large, complex, and dynamic. Just as multiple disciplines may be required, the nature of global health issues also often leads to multiple actors using a variety of means to achieve different goals. Although the composition, funding mechanisms, values and goals of actors are important to the study and practice of global health [30], currently any individual or group can be an agent of global health. The all-inclusive nature of the work means that defining actors is not essential to the definition, though it is part of specifying means and solutions.

#### 4. Reactive/proactive

Determining whether the provision of global health should be reactive, proactive or a combination of both depends on whether the focus is put upon current crises or future events that may result in crises. In a reactive approach, we respond to issues already at a crisis point when harm is likely already occurring and immediate solutions, such as famine relief, are required. A proactive response involves more foresight, for example, devising crop varieties adapted for climate change [4]. Determining a reactive, proactive or blended approach to action on a global health problem will direct resources to the most appropriate mix of solutions. These characteristics are descriptive of the means, the solutions, and the approach that is taken by global health actors; but are not descriptive of the field, and therefore not an essential part of the definition.

While it would be difficult to reach agreement upon a single, international definition of global health, nation-level common definitions could assist in anticipating and coordinating strategies and initiatives across regions and sectors. The European Union has identified and communicated their definition of global health [13] and the Expert Panel on Canada’s Strategic Role in Global Health selected the Koplan et al. [14] definition as the base for decision making in Canada.

Now that this definition is in place, it can provide direction to academics and organizations working in the field. A different choice would have significantly altered the practice of global health in Canada from the path set by the chosen definition. For example, by picking a definition that includes equity, it indicates that under Canadian global health strategies equity is an essential component. Had the expert panel selected the Brown definition [12], which does not include equity, it would have indicated that equity was not a primary concern from the Canadian perspective on global health. This would have had enormous implications for the future practice and study of global health in Canada.

With the chosen Canadian definition for global health the expert panel has provided the international global health community, researchers and policy makers an indication of future directions for Canadian global health initiatives. It would benefit the international global health community to have all international actors working in the field clearly indicate their own understanding of the term global health and the definition that frames their work.
